# Supplementary material for: Influence of age on gadoxetic acid disodium-induced transient respiratory motion artifacts in pediatric liver MRI
Source: PLoS One. 2022 Mar 2;17(3):e0264069. doi: 10.1371/journal.pone.0264069 (PMC8890729; doi:10.1371/journal.pone.0264069)
Supplement: S1 Table — (DOCX) [file pone.0264069.s001.docx]

| **S1 Table.** Liver MR scan protocol with Gd-EOB-DTPA | | | | | | | | | | |
| --- | --- | --- | --- | --- | --- | --- | --- | --- | --- | --- |
| **Sequences** | **Slice  orientation** | **Matrix** | **Voxel mm** | **FOV mm** | **SL mm** | **gap mm** | **TR**  **Ms** | **TE ms** | **FA**  **Degree** | **Time s** |
| GRE T1 (2D flash) in-phase* | Axial | 320x320 | 0.5x0.5x5 | 350 | 5 | 1 | 130 | 2.38 | 70 | 21 |
| GRE T1 (2D flash) opposed phase* | Axial | 320x320 | 0.5x0.5x5 | 350 | 5 | 1 | 130 | 4.85 | 70 | 21 |
| T2 HASTE FatSat thick radial* | Coronal | 384x384 | 0.8x0.8x50 | 300 | 50 | 25 | 4500 | 750 | 180 | 62 |
| MRCP sequence with MIP* | Coronal | 256x256 | 1.1x1.1x1 | 280 | 1 |  | 2000 | 703 | 140 | 235 |
| T2 HASTE* | Axial | 320x320 | 1.1x1.1x5 | 350 | 5 | 1 | 1000 | 95 | 160 | 47 |
| T1 VIBE FatSat non-contrast | Axial | 320x320 | 1.1x1.1x2.5 | 350 | 2.5 | 0.5 | 4.76 | 2.29 | 10 | 16 |
| T1 VIBE FatSat dynamics (arterial and portal-venous) | Axial | 320x320 | 1.1x1.1x2.5 | 350 | 2.5 | 0.5 | 4.76 | 2.29 | 10 | 16x3 |
| T1 VIBE FatSat 5 min post contrast (equilibrium) | Coronal | 288x288 | 1.3x1.3x2.2 | 380 | 2.2 | 0.44 | 2.97 | 1.38 | 10 | 16 |
| T1 VIBE FatSat 5 min post contrast (equilibrium) | Axial | 320x320 | 1.1x1.1x2.5 | 350 | 2.5 | 0.5 | 4.76 | 2.29 | 10 | 16 |
| T2 HASTE FatSat* | Axial | 320x320 | 1.1x1.1x5 | 350 | 5 | 1 | 1000 | 95 | 160 | 47 |
| T2 HASTE* | coronal | 256x256 | 1.4x1.4x4.5 | 350 | 4.5 | 0.9 | 1000 | 102 | 180 | 45 |
| DWI TSE-EP/ADC* | Axial | 192x192 | 1.8x1.8x5 | 350 | 5 | 1 | 5100 | 67 |  | 133 |
| T2 TSE FatSat triggered* | Axial | 384x384 | 0.9x0.9x5 | 350 | 5 | 1 | 2000 | 105 | 160 | 260 |
| T1 VIBE FatSat 20 min post contrast (hepatobiliary)* | Axial | 320x320 | 1.1x1.1x2.5 | 350 | 2.5 | 0.5 | 4.76 | 2.29 | 10 | 16 |
| T1 VIBE FatSat 20 min post contrast (hepatobiliary)* | Coronal | 288x288 | 1.3x1.3x2.2 | 380 | 2.2 | 0.44 | 2.97 | 1.38 | 10 | 16 |
| HASTE = Half-Fourier Acquisition Single-shot Turbo spin Echo imaging; DWI TSE-EP/ADC = Diffusion Weighted Imaging Turbo Spin Echo-Echo-Planar; MRCP= Magnetic resonance cholangiopancreatography; MIP=Maximum intensity projection; GRE = Gradient echo; VIBE = Volumetric interpolated breath-hold examination; FOV = field of view; Voxel = voxel size; SL = slice thickness; TR = repetition time; TE = echo time; FA = flip angle; Time = acquisition time. *not evaluated for the study | | | | | | | | | | |
