# Supplementary material for: Influence of age on gadoxetic acid disodium-induced transient respiratory motion artifacts in pediatric liver MRI
Source: PLoS One. 2022 Mar 2;17(3):e0264069. doi: 10.1371/journal.pone.0264069 (PMC8890729; doi:10.1371/journal.pone.0264069)
Supplement: S2 Table — (DOCX) [file pone.0264069.s002.docx]

| **S2 Table.** Indications for liver MRI | |
| --- | --- |
| **Indication** | **No. of Gd-EOB-DTPA MRIs** |
| Transplantation | 23 (10) |
| - Liver transplantation | 20 |
| - Liver and kidney transplantation | 2 |
| - GvHD | 1 |
| Masses | 73 (32) |
| - Intrahepatic mass | 53 |
| - Extrahepatic mass | 21 |
| Pancreaticobiliary disorders | 50 (22) |
| - Cholecystolithiasis, choledocholithiasis | 10 |
| - Biliary atresia | 5 |
| - Caroli disease and choledochal cyst | 4 |
| - Cholangitis | 1 |
| - Pancreas or liver trauma | 7 |
| - Pancreatitis | 23 |
| Hepatobiliary functional disorders | 81 (36) |
| - Virus-Hepatitis, AIH, PBC, PSC | 18 |
| - Metabolic disease | 15 |
| - Cystic fibrosis | 9 |
| - IBD with hepatic dysfunction | 20 |
| - Hepatopathy and portal hypertension | 19 |
| Data presented are absolute numbers and percentages in parentheses apply to the entire cohort. GvHD = graft versus host disease; AIH = autoimmune hepatitis; PBC = primary biliary cholangitis; PSC = primary sclerosing cholangitis; IBD = inflammatory bowel disease | |
