# Supplementary material for: Influence of age on gadoxetic acid disodium-induced transient respiratory motion artifacts in pediatric liver MRI
Source: PLoS One. 2022 Mar 2;17(3):e0264069. doi: 10.1371/journal.pone.0264069 (PMC8890729; doi:10.1371/journal.pone.0264069)
Supplement: S3 Table — (DOCX) [file pone.0264069.s003.docx]

| **S3 Table.** Patients with TSM events after i.v. application of Gd-EOB-DTPA-MRI | | | | | | | |
| --- | --- | --- | --- | --- | --- | --- | --- |
| Patient Nr. | Number of Gd-EOB-DTPA-MRI | Number of TSM events | At which MRI did the first TSM event occur? | At which MRI did the second TSM event occur? | Uneventful subsequent Gd-EOB-DTPA-MRI after a TSM event | Age at the first TSM event | Age at the second TSM event |
| 20 | 1 | 1 | 1^st^ | - | - | 14.0 | - |
| 26 | 1 | 1 | 1^st^ | - | - | 18.0 | - |
| 39 | 1 | 1 | 1^st^ | - | - | 15.0 | - |
| 46 | 1 | 1 | 1^st^ | - | - | 13.0 | - |
| 54 | 1 | 1 | 1^st^ | - | - | 10.0 | - |
| **57** | **6** | **1** | **1^st^** | - | **Yes** | **11.0** | - |
| **63** | **2** | **1** | **1^st^** | - | **Yes** | **14.0** | - |
| 64 | 1 | 1 | 1^st^ | - | - | 17.0 | - |
| 69 | 1 | 1 | 1^st^ | - | - | 16.0 | - |
| **72** | **4** | **1** | **3^rd^** | - | **Yes** | **11.0** | - |
| 77 | 1 | 1 | 1^st^ | - | - | 14.0 | - |
| 79 | 1 | 1 | 1^st^ | - | - | 16.0 | - |
| 88 | 1 | 1 | 1^st^ | - | - | 15.0 | - |
| **97** | **2** | **1** | **2^nd^** | - | - | 15.0 | - |
| **99** | **4** | **1** | **3^rd^** | - | **Yes** | **11.0** | - |
| 101 | 1 | 1 | 1^st^ | - | - | 17.0 | - |
| **111** | **6** | **2** | **1^st^** | **3^rd^** | **Yes** | **16.0** | **16.0** |
| 117 | 1 | 1 | 1^st^ | - | - | 13.0 | - |
| **124** | **6** | **1** | **6^th^** | **-** | **-** | 11.0 | - |
| **130** | **2** | **1** | **2^nd^** | **-** | **-** | 16.0 | - |
| **131** | **2** | **1** | **2^nd^** | **-** | **-** | 13.0 | - |
| 135 | 1 | 1 | 1^st^ | - | - | 11.0 | - |
| 146 | 1 | 1 | 1^st^ | - | - | 12.0 | - |
